# Achievement goal orientations and digital literacy among primary and secondary school physical education teachers: the serial mediating roles of person–organization fit and positive coping

**DOI:** 10.3389/fpubh.2026.1959121

**Published:** 2026-10-06

**Authors:** Dongbin Lai, Shufeng Zhang

**Affiliations:** 1 School of Physical Education, Guangzhou Huali College, Jiangmen, China; 2 Division of Sports Science and Physical Education, Tsinghua University, Beijing, China

**Keywords:** achievement goal orientations, digital literacy, PE teachers, person–organization fit, positive coping

## Abstract

**Introduction:**

Teachers play a pivotal role in advancing educational digital transformation, and the digital literacy (DL) of primary and secondary school physical education (PE) teachers is crucial to the effective integration of digital technologies into physical education. This study examined the relationships between achievement goal orientations (AGOs) and digital literacy among primary and secondary school physical education teachers. It further investigated the specific mediating effects and serial mediating effects of person–organization fit (POF) and positive coping (PC) in these relationships.

**Methods:**

A questionnaire survey was conducted with 1,654 primary and secondary school physical education teachers. Data were collected using the Teachers’ Achievement Goals in Work Questionnaire, the Perceived Person–Organization Fit Scale, the Positive Coping Subscale, and the Digital Literacy Scale for Primary and Secondary School Physical Education Teachers. SPSS 26.0 was used to conduct descriptive statistics, correlation analyses, and tests for common method bias. Structural equation modeling (SEM) was conducted using AMOS 29.0. Mastery goals (MGs), performance-approach goals (PAGs), and performance-avoidance goals were simultaneously entered into the model to examine their independent effects on digital literacy and to test the serial mediating effects of person–organization fit and positive coping. The indirect effects were examined using the bootstrap method with 5,000 resamples.

**Results:**

First, mastery goals and performance-approach goals significantly and positively predicted teachers’ digital literacy, whereas the direct effect of performance-avoidance goals on digital literacy was not significant. Second, mastery goals, performance-approach goals, and performance-avoidance goals were each indirectly associated with digital literacy through person–organization fit. Third, mastery goals and performance-approach goals showed significant indirect effects on digital literacy through positive coping, whereas the mediating effect of positive coping in the relationship between performance-avoidance goals and digital literacy was not significant. Fourth, person–organization fit and positive coping exerted significant serial mediating effects in the relationships between each achievement goal orientation and digital literacy. In particular, the association between performance-avoidance goals and digital literacy was primarily transmitted through the indirect pathways involving person–organization fit and positive coping.

**Discussion:**

Achievement goal orientations represent important individual-level factors associated with the digital literacy of primary and secondary school physical education (PE) teachers, while person–organization fit and positive coping constitute important organizational and psychological mechanisms underlying these associations. Schools should guide teachers toward adaptive achievement goals; strengthen institutional support for digital teaching; enhance teachers’ fit with organizational values, requirements, and resources; and cultivate their capacity to cope positively with digital teaching challenges. These efforts may contribute to the development of teachers’ digital literacy and the effective digital transformation of physical education.

## 1 Introduction

Digital technologies are increasingly reshaping education and have become a major driver of high-quality educational development worldwide. The report of the 20th National Congress of the Communist Party of China called for advancing educational digitalization and building a learning society and a learning nation committed to lifelong learning. During the fifth group study session of the Political Bureau of the CPC Central Committee, President Xi Jinping further emphasized that educational digitalization represents a critical breakthrough for opening new pathways and creating new advantages in educational development. Educational digitalization has, therefore, become not only an essential component of contemporary educational development but also a major avenue for educational reform and pedagogical innovation. Digital literacy (DL) provides a fundamental basis for the successful digital transformation of education. As frontline practitioners of this transformation, PE teachers play a central role in integrating digital technologies into the Physical Education and Health curriculum. Their level of DL influences the extent to which they can participate actively, proactively, and creatively in digitally supported teaching practices. It also constitutes an important professional capacity underpinning the sustained and effective development of digital education. Accordingly, strengthening the DL of PE teachers has become an urgent priority in the context of educational digital transformation.

Physical education is characterized by open instructional spaces, continuously changing student movement states, relatively large class sizes, and substantial safety risks. Consequently, physical education teachers already face considerable challenges related to classroom organization, the accommodation of individual differences, the precise regulation of exercise intensity, and the objective assessment of student learning. Although digital technologies offer new possibilities for movement analysis, physical fitness monitoring, and personalized instruction, they also create additional demands, including learning to operate intelligent devices, selecting appropriate digital resources, collecting and analyzing movement data, and developing digitally enriched instructional content (1). When teachers have limited competence in applying digital technologies, and schools provide insufficient equipment, training, or organizational support, these additional demands may further increase teachers’ workload and weaken their willingness and sustained engagement in digital teaching practices. Previous studies have shown that teachers’ DL is shaped not only by technological conditions but also by individual motivation, attitudes, and organizational support (2). Nevertheless, the internal mechanisms through which these factors contribute to the development of teachers’ DL remain insufficiently understood.

Person–organization fit (POF) theory provides a useful framework for explaining the development of physical education teachers’ DL. The theory proposes that the degree of compatibility between individuals and their organizations influences how they evaluate organizational environments and how they subsequently behave within those environments (3). A high level of POF can reduce role conflict, enhance psychological comfort, and strengthen organizational identification (4), thereby providing a psychological foundation for teachers to cope proactively with the challenges associated with educational digital transformation. Achievement goals, as motivational orientations, also shape individuals’ willingness to invest effort in tasks (5) and influence their perceptions of compatibility with the surrounding environment (6). However, limited research has systematically examined how achievement goal orientations (AGOs) influence physical education teachers’ DL through the joint roles of POF and positive coping (PC) in the context of digital transformation. This issue is particularly important for primary and secondary school PE teachers. Due to the openness, dynamism, and practical nature of PE, the application of digital technologies requires teachers not only to acquire relevant technological skills but also to continuously adjust their teaching philosophies and practices. Therefore, the development of PE teachers’ DL is influenced not only by external environmental factors but is also closely associated with their motivational characteristics and their cognitive and behavioral responses to digital transformation. As an important individual motivational characteristic, AGOs may influence how teachers evaluate digital transformation demands and determine corresponding behavioral strategies. Examining AGOs only as an overall construct may obscure the differentiated mechanisms through which different motivational orientations contribute to DL development. Therefore, it is necessary to investigate the pathways through which different AGOs influence PE teachers’ DL, thereby further elucidating the psychological and behavioral mechanisms underlying the development of DL across distinct achievement motivation orientations. Drawing on POF theory and achievement goal theory, this study investigates the psychological and behavioral mechanisms underlying the development of DL among PE teachers. Specifically, it examines whether POF and PC independently and sequentially mediate the relationships between different AGOs and teachers’ DL. The findings are expected to extend current understanding of the motivational and organizational antecedents of teacher DL and provide theoretical and practical implications for enhancing the digital competence of physical education teachers.

### 1.1 Achievement goal orientations and digital literacy

AGOs refer to the purposes or reasons underlying individuals’ pursuit of achievement-related tasks. They reflect individuals’ underlying value orientations toward achievement and shape their cognition, emotions, and behavioral strategies (7). The achievement goal framework generally distinguishes among mastery goals (MGs), performance-approach goals (PAGs), and performance-avoidance goals (PAvGs) (8). First, MGs emphasize competence development through the acquisition of knowledge and improvement of ability. Teachers with strong MGs tend to place greater value on professional growth and are more likely to interpret difficulties encountered in using digital technologies as opportunities for learning and development. Accordingly, they may actively acquire knowledge related to digital teaching, experiment with digital tools, and continuously adjust their technology-use strategies in response to classroom experience. Previous research has shown that mastery-oriented individuals are more likely to use digital tools to acquire knowledge and improve work quality while maintaining a critical awareness of technology use (9). Second, PAGs emphasize demonstrating competence through favorable comparisons with others. Individuals with strong PAGs tend to be particularly attentive to how colleagues, students, and school administrators evaluate their teaching performance (10). In the context of educational digital transformation, digital teaching competence has increasingly become an important indicator of teachers’ professional performance. These teachers may therefore be motivated to learn and apply digital technologies to demonstrate their instructional competence, gain organizational recognition, or maintain a competitive advantage, thereby contributing to the development of their DL. Third, PAvGs are characterized by efforts to avoid unfavorable judgments of one’s competence, often through the selection of relatively simple or low-risk tasks. Teachers with strong PAvGs may worry about making operational errors or revealing insufficient competence when using digital technologies. As a result, they may prefer less challenging teaching tasks and avoid unfamiliar digital tools or digitally supported instructional approaches. Performance-avoidance-oriented individuals have been found to use digital media less frequently for academic purposes, hold more negative attitudes toward learning, and regulate their behavior primarily in response to anticipated negative outcomes, which may produce maladaptive consequences (11). Over time, such avoidance may reduce teachers’ opportunities to engage with, learn, and practice digital technologies, thereby constraining the development of their digital knowledge, application competence, and innovative awareness.

Based on the above reasoning, the following hypothesis is proposed:

*H1a:* MG positively predict DL among primary and secondary school physical education teachers.

*H1b:* PAG positively predict DL among primary and secondary school physical education teachers.

*H1c:* PAvG negatively predict DL among primary and secondary school physical education teachers.

### 1.2 The mediating role of person–organization fit

POF refers to the compatibility between an individual and an organization. It is commonly conceptualized in terms of value congruence, demands–abilities fit, and needs–supplies fit. Fit arises when one party satisfies the needs of the other, when the individual and organization share similar fundamental characteristics, or when both conditions are present (12). On the one hand, MGs have been positively associated with perceived POF (13). Individuals with strong MGs tend to demonstrate greater cognitive flexibility and more adaptive behavior, enabling them to adjust their actions and strategies in response to changes in their environment and workplace and thereby better meet organizational requirements (14). Empirical research has also found a significant positive relationship between performance goal orientations and POF. Individuals with such orientations may seek positive evaluations and demonstrate their competence as a means of becoming more effectively integrated into the organization (15). On the other hand, value congruence between teachers and schools may strengthen teachers’ identification with their schools and foster more favorable attitudes toward organizational digital transformation, thereby encouraging them to improve their DL in practice. Needs–supplies fit reflects the extent to which schools provide teachers with the resources required for digital teaching, including technological equipment, professional training opportunities, and financial support. Demands–abilities fit, by contrast, captures the compatibility between teachers’ digital capabilities and schools’ expectations for digitally supported instruction. A high level of demands–abilities fit indicates that teachers possess the competence required to use digital technologies effectively to improve classroom teaching, which itself constitutes an important dimension of DL.

Accordingly, the following hypothesis is proposed: H2. POF mediates the relationships between AGOs and the DL of PE teachers.

### 1.3 The mediating role of positive coping

PC refers to a set of behavioral strategies through which individuals actively address stress and difficulties by means of cognitive adjustment, problem solving, and support seeking (16). On the one hand, AGOs shape how individuals respond to challenges. Teachers with strong MGs emphasize competence development and are therefore more likely to address difficulties in digital teaching through self-regulation, feedback seeking, and continuous learning (17). Teachers with strong PAGs focus on demonstrating competence and obtaining favorable evaluations. They may therefore participate in digital technology learning and application in response to external recognition and performance expectations, although their willingness to seek help proactively and sustain innovative behavior may depend on the specific context (18). On the other hand, educational digital transformation requires PE teachers to address new demands related to digital tool operation, instructional resource development, movement-data analysis, and digitally supported lesson design (19). Teachers who adopt PC strategies are more likely to address difficulties in technology use through active learning, support seeking from colleagues and organizations, and sustained practice. Such behaviors facilitate the accumulation of digital knowledge and skills and support the continued integration of digital technologies into teaching (20).

Based on this reasoning, the following hypothesis is proposed: H3. PC mediates the relationships between AGOs and the DL of PE teachers.

### 1.4 The serial mediating roles of person–organization fit and positive coping

POF emphasizes the compatibility between individuals’ values and needs and an organization’s values, culture, and goals (21). A high level of fit can strengthen organizational identification, belongingness, and adaptability, making individuals more likely to develop positive work attitudes and engage in proactive behavior (22). Research has shown that when individuals and organizations share similar values and developmental goals, individuals are more likely to respond to organizational change in positive and adaptive ways (23). According to self-determination theory, the satisfaction of individuals’ needs for autonomy, competence, and relatedness facilitates intrinsic motivation and positive behavior (24). A high level of POF may strengthen employees’ sense of belonging and organizational identification (25), satisfy their needs for competence development (26), and enhance their sense of autonomy through organizational trust and support (27). Furthermore, person–organization fit and positive coping represent distinct psychological processes in the proposed mechanism. Person–organization fit mainly reflects individuals’ cognitive evaluation of the compatibility between themselves and their organizational environment, whereas positive coping represents behavioral strategies adopted when individuals encounter specific challenges. Therefore, POF primarily reflects how individuals understand and evaluate their organizational environment, whereas PC represents the behavioral responses individuals adopt based on such evaluations. According to cognitive appraisal theory, individuals generally evaluate the relationship between environmental demands and personal resources before selecting coping responses (28). This theoretical perspective provides a rationale for the potential sequential relationship between POF and PC: Individuals first develop cognitive evaluations regarding the extent to which they fit with their organizational environment and subsequently select corresponding coping strategies based on these evaluations. Therefore, in the context of school digital transformation, primary and secondary school physical education teachers may first assess whether their abilities, values, and developmental needs are aligned with the school’s digital development goals, technological requirements, and available resources. Such evaluations may then influence whether they adopt PC strategies, such as proactive learning, problem solving, and seeking support. Ultimately, PC may further contribute to the development of DL by facilitating teachers’ acquisition of digital technology knowledge, skill practice, and innovative application.

Accordingly, the following hypothesis is proposed: H4. POF and PC sequentially mediate the relationships between AGOs and the DL of PE teachers.

The hypothesized research model is presented in Figure 1.

**Figure 1:**
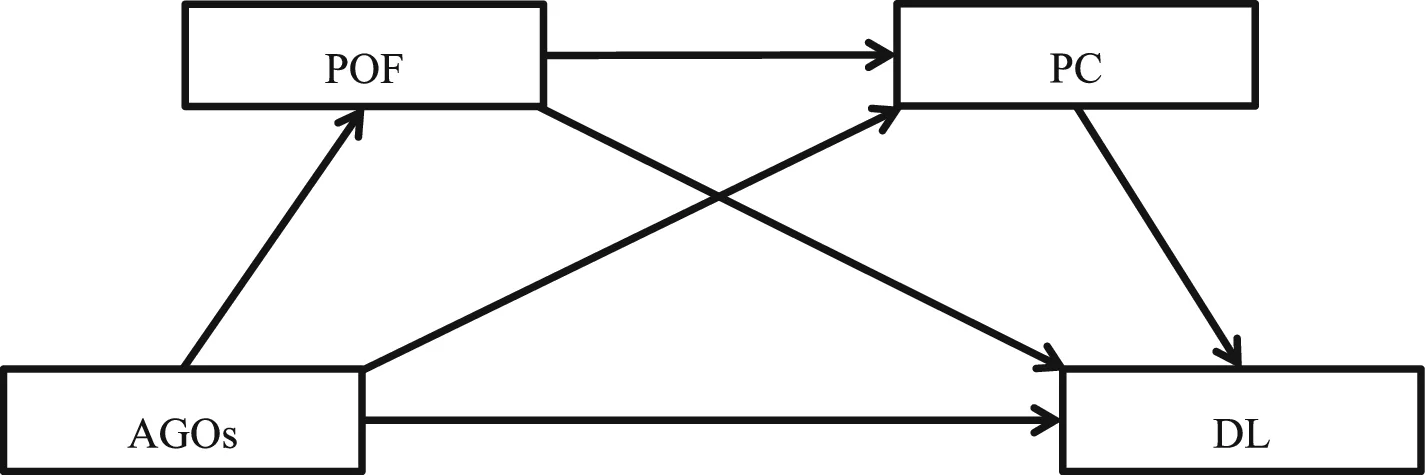
Hypothesized research model.

## 2 Methods

### 2.1 Participants and procedure

Convenience sampling was used to recruit PE teachers from cities and districts across Guangdong Province, China. The questionnaires were distributed online through Wenjuanxing, a widely used Chinese survey platform. A total of 1,967 questionnaires were collected in this study. To ensure data quality, the collected questionnaires were rigorously screened according to predefined data-cleaning criteria. Invalid questionnaires were excluded if they involved duplicate responses, patterned responding, logical inconsistencies, or unrealistically short completion times. Finally, 1,654 valid questionnaires were retained, yielding an effective response rate of 84.09%. The final sample comprised 999 male teachers (60.40%) and 655 female teachers (39.60%). Regarding age, 173 participants (10.46%) were aged 25 years or younger, 240 (14.51%) were aged 26–30 years, 329 (19.89%) were aged 31–35 years, 374 (22.61%) were aged 36–40 years, 314 (18.98%) were aged 41–45 years, and 224 (13.54%) were aged 46 years or older. Regarding monthly income, 254 participants (15.36%) earned less than RMB 3,000, 393 (23.76%) earned RMB 3,000–4,999, 494 (29.87%) earned RMB 5,000–6,999, 416 (25.15%) earned RMB 7,000–8,999, and 97 (5.86%) earned RMB 9,000 or more. The study protocol was reviewed and approved by the Human Research Ethics Committee of Guangzhou Sport University (Approval No. 2024LCLL-71). Additional demographic information is presented in Table 1.

**Table 1:** Demographic characteristics of participants (*N* = 1,654).

Category	Group	*n*	%
Region	Pearl River Delta	573	34.64
	Eastern Guangdong	434	26.24
	Western Guangdong	432	26.12
	Northern Guangdong	215	13.00
Teaching stage	Primary school	725	43.83
	Secondary school	929	56.17
School location	Urban area	795	48.07
	County/town area	532	32.16
	Rural area	327	19.77

### 2.2 Measures

#### 2.2.1 Digital literacy of primary and secondary school physical education teachers

DL was assessed using the DL Scale for PE Teachers developed by Xu (29). The scale consists of 41 items across five dimensions: digital awareness, comprising nine items (e.g., “I actively attempt to introduce emerging technologies, such as wearable devices and AI coaching systems, into my classes to address limitations in traditional teaching”); digital technology knowledge and skills, comprising seven items (e.g., “I understand the core functions of video analysis software commonly used in physical education, such as slow-motion playback and motion-tracking tools”); digital application, comprising 15 items (e.g., “I can proficiently use augmented reality, virtual reality, or slow-motion playback applications to provide movement demonstrations for students”); digital social responsibility, comprising four items (e.g., “Before using video recordings or wearable devices to collect students’ physical activity data, I clearly explain the purpose and scope of data collection and obtain informed consent”); and digital professional development, comprising six items (e.g., “I actively participate in or lead research projects related to the digital transformation of physical education to explore solutions to practical teaching problems”). All items were rated on a 5-point Likert scale ranging from 1 (strongly disagree) to 5 (strongly agree). Higher scores indicated higher levels of DL. In this study, the scale demonstrated excellent internal consistency, with a Cronbach’s α of 0.965.

#### 2.2.2 Achievement goal orientations

AGOs were measured using the Teachers’ Achievement Goals in Work Questionnaire developed by Papaioannou and Christodoulidis (30). The scale has been translated into Chinese and subsequently applied and validated among occupational groups in China, showing satisfactory construct validity and internal consistency (31). The questionnaire comprises 12 items across three dimensions: MGs, comprising four items (e.g., “I intend to try even harder to learn new things related to what I teach”); PAGs, comprising four items (e.g., “I will always try to outperform my colleagues”); and PAvGs, comprising four items (e.g., “I want to avoid teaching content in which I may appear incapable”). Items were rated on a 5-point Likert scale. Higher scores on each subscale indicated a stronger orientation toward the corresponding achievement goal. In this study, the overall Cronbach’s α for the questionnaire was 0.848.

#### 2.2.3 Person–organization fit

POF was assessed using the nine-item scale developed by Cable and DeRue (32). The scale was translated and culturally adapted by Cao Yang and has been subsequently applied and validated among Chinese populations, demonstrating satisfactory construct validity and internal consistency (33). The scale comprises three dimensions: value congruence, consisting of three items (e.g., “My personal values match my organization’s values and culture”); demands–abilities fit, consisting of three items (e.g., “My abilities and training are a good fit with the requirements of my job”); and needs–supplies fit, consisting of three items (e.g., “There is a good fit between what my job offers me and what I am looking for in a job”). All items were rated on a 5-point Likert scale, with higher scores indicating a higher level of perceived POF. In this study, the scale showed good internal consistency, with a Cronbach’s α of 0.872.

#### 2.2.4 Positive coping

PC was measured using the PC Subscale of the Simplified Coping Style Questionnaire developed by Xie (34). The subscale comprises 12 items, including statements such as “Drawing on how others have dealt with similar difficult situations.” Items were rated on a 4-point scale ranging from 0 (never adopted) to 3 (frequently adopted), with scores of 1 and 2 representing occasionally adopted and sometimes adopted, respectively. Higher scores indicated a greater tendency to use PC strategies. In this study, the subscale demonstrated good internal consistency, with a Cronbach’s α of 0.887.

### 2.3 Data analysis

All statistical analyses were performed using IBM SPSS Statistics 26.0, IBM SPSS Amos 29.0, and the PROCESS macro developed by Hayes. First, Harman’s single-factor test was performed in SPSS 26.0 to assess potential common method bias. A single-factor confirmatory factor analysis was subsequently performed in Amos 29.0 as an additional test of common method bias. Model fit was evaluated using the chi-square-to-degrees-of-freedom ratio (χ^2^/df), the root mean square error of approximation (RMSEA), the non-normed fit index (NNFI), the comparative fit index (CFI), and the standardized root mean square residual (SRMR). Based on conventional criteria, model fit was considered acceptable when χ^2^/df was below 3.00, RMSEA was below 0.08, NNFI and CFI were above 0.90, and SRMR was below 0.05. Furthermore, standardized factor loadings, composite reliability (CR), and average variance extracted (AVE) were used to assess the convergent validity of the measurement model. Discriminant validity was examined by comparing the correlations among latent constructs with the square roots of their corresponding AVE values. Subsequently, Pearson’s correlation analyses were conducted to examine the relationships among the study variables. Finally, structural equation modeling (SEM) was conducted to test the hypothesized model. Unlike establishing three separate mediation models, this study simultaneously included mastery goals, performance-approach goals, and performance-avoidance goals in the same structural model to control for the shared variance among different achievement goal orientations and estimate their independent effects on digital literacy. In the structural model, person–organization fit and positive coping were specified as serial mediators, forming a serial mediation model of “achievement goal orientations → person–organization fit → positive coping → digital literacy.” The bootstrap method was used to examine the indirect effects with 5,000 resamples, and the direct effects, indirect effects, and their 95% confidence intervals (CIs) were reported. An indirect effect was considered statistically significant when the bootstrap confidence interval did not include zero.

## 3 Results

### 3.1 Preliminary analysis and measurement model assessment

Because all variables were assessed using self-report measures, common method variance was a potential concern. Several procedural remedies were implemented during data collection. Specifically, participants were informed in the survey instructions that their responses would remain anonymous and that the data would be used exclusively for scientific research. Harman’s single-factor test was first conducted to assess potential common method bias. The unrotated exploratory factor analysis extracted 10 factors with eigenvalues greater than 1, and the first factor accounted for 30.465% of the total variance, which was below the commonly used threshold of 40%. A single-factor confirmatory factor analysis was subsequently performed by loading all items onto one latent factor. The single-factor model showed poor overall fit to the data: χ^2^/df = 8.413, CFI = 0.665, TLI = 0.655, IFI = 0.665, RMSEA = 0.067, and SRMR = 0.076. These results indicate that the covariance structure among the study variables cannot be adequately explained by a single latent factor. However, it should be noted that the single-factor CFA can only serve as an auxiliary assessment of common method bias and cannot completely rule out the possibility of common method variance.

Subsequently, CFA was conducted to examine the measurement structure and construct validity of the study variables. Considering that achievement goal orientation consists of three theoretically distinct dimensions—mastery goals, performance-approach goals, and performance-avoidance goals—these dimensions were specified as separate latent constructs. Together with POF, PC, and DL, a six-factor measurement model was established. The results indicated that the six-factor measurement model demonstrated an acceptable fit to the data (χ^2^ = 7205.34, df = 2,612, χ^2^/df = 2.76, RMSEA = 0.033, SRMR = 0.029, TLI = 0.921, CFI = 0.926). All fit indices met the recommended criteria, indicating that the proposed measurement model adequately represented the latent structure of the study variables. Therefore, the six-factor model was retained for subsequent structural model analyses. The convergent validity of the measurement model was further examined. The results showed that all standardized factor loadings exceeded 0.50. Specifically, the factor loadings ranged from 0.68 to 0.82 for mastery goals, 0.71 to 0.84 for performance-approach goals, 0.66 to 0.81 for performance-avoidance goals, 0.63 to 0.79 for POF, 0.61 to 0.80 for PC, and 0.60 to 0.83 for DL. These results indicated that all measurement indicators adequately represented their corresponding latent constructs. Furthermore, composite reliability (CR) and average variance extracted (AVE) were calculated to assess the convergent validity of each construct. The results showed that CR values for all latent constructs ranged from 0.84 to 0.96, exceeding the recommended threshold of 0.70. Meanwhile, AVE values ranged from 0.54 to 0.64, all above the recommended criterion of 0.50, indicating satisfactory internal consistency and convergent validity. Finally, discriminant validity was assessed by comparing the correlations among latent constructs with the square roots of their corresponding AVE values. The results showed that the correlations among the latent constructs ranged from 0.32 to 0.64, all of which were lower than the corresponding square roots of AVE. These findings suggest that although the six constructs were correlated, they remained empirically distinguishable. Therefore, the measurement model demonstrated satisfactory construct validity and provided a reliable basis for further examining the mechanisms through which AGOs influence DL.

### 3.2 Descriptive statistics and correlations

Pearson’s correlation analyses were performed to examine the relationships among the study variables, and the results are presented in Table 2. MG, PAG, PAvG, POF, PC, and DL were all significantly and positively correlated, with correlation coefficients ranging from 0.204 to 0.575 (all *p*s < 0.001). The strongest correlation was observed between MG and POF (*r* = 0.575, *p* < 0.001). POF was also strongly and positively associated with DL (*r* = 0.550, *p* < 0.001) and PC (*r* = 0.544, *p* < 0.001). In addition, PC was significantly and positively correlated with DL (*r* = 0.531, *p* < 0.001). Monthly income showed a weak positive correlation with PC (*r* = 0.052, *p* < 0.05). By contrast, gender and age were not significantly correlated with any of the core study variables.

**Table 2:** Descriptive statistics and correlations among the study variables.

Variable	M	SD	1	2	3	4	5	6	7	8	9
1. Gender	-	-	1								
2. Age	36.484	8.067	−0.002	1							
3. Monthly income	-	-	−0.017	0.702***	1						
4. MG	3.904	0.858	0.008	0.010	0.012	1					
5. PAG	3.509	0.920	−0.029	0.002	0.002	0.406***	1				
6. PAvG	3.236	0.936	−0.026	0.015	0.022	0.212***	0.448***	1			
7. POF	3.719	0.773	−0.004	0.011	0.025	0.575***	0.478***	0.311***	1		
8. PC	1.968	0.660	0.005	0.015	0.052*	0.493***	0.383***	0.216***	0.544***	1	
9. DL	3.641	0.716	0.009	−0.017	0.028	0.508***	0.357***	0.204***	0.550***	0.531***	1

* *p* < 0.05, ** *p* < 0.01, *** *p* < 0.001, *N* = 1654.

### 3.3 Mechanisms underlying the association between achievement goal orientations and digital literacy

To further examine the mechanisms underlying the effects of different achievement goal orientations on digital literacy among primary and secondary school physical education teachers and to avoid potential bias caused by the shared variance among different goal orientations, a joint structural equation model was constructed. Specifically, MGO, PAPG, and PAVG were simultaneously included in the model for analysis. Based on person–organization fit theory and positive coping theory, this study further examined the roles of POF and PC in the association between achievement goal orientations and DL. A serial mediation model of “achievement goal orientations → POF → PC → DL” was constructed. Parameters were estimated using the maximum likelihood estimation method, and the indirect effects were tested using the bootstrap method with 5,000 resamples.

#### 3.3.1 Direct effects in the joint structural model

The path analysis results of the joint structural model are presented in Table 3. The results showed that, after simultaneously controlling for the three achievement goal orientations, all three goal orientations significantly and positively predicted POF. Among them, MGO showed the strongest predictive effect on POF (*β* = 0.452, *p* < 0.001), followed by PAPG (*β* = 0.248, *p* < 0.001) and PAVG (*β* = 0.103, *p* < 0.001).

**Table 3:** Standardized direct effects of the joint structural equation model.

Effect pathway	*β*	*p*
MGO → POF	0.452	<0.001
PAPG → POF	0.248	<0.001
PAVG → POF	0.103	<0.001
MGO → PC	0.246	<0.001
PAPG → PC	0.117	<0.001
PAVG → PC	0.004	0.853
POF → PC	0.345	<0.001
MGO → DL	0.205	<0.001
PAPG → DL	0.144	<0.001
PAVG → DL	0.001	0.961
POF → DL	0.264	<0.001
PC → DL	0.270	<0.001

Further analysis indicated that POF significantly and positively predicted PC (*β* = 0.345, *p* < 0.001). In addition, MGO (*β* = 0.246, *p* < 0.001) and PAPG (*β* = 0.117, *p* < 0.001) significantly and positively predicted PC, whereas the predictive effect of PAVG on PC was not significant (*β* = 0.004, *p* = 0.853).

Regarding DL, after simultaneously including the three achievement goal orientations and the mediating variables, MGO (*β* = 0.205, *p* < 0.001) and PAPG (*β* = 0.144, *p* < 0.001) remained significant positive predictors of DL. In contrast, the direct effect of PAVG on DL was not significant (*β* = 0.001, *p* = 0.961). Furthermore, both POF (*β* = 0.264, *p* < 0.001) and PC (*β* = 0.270, *p* < 0.001) significantly and positively predicted DL.

#### 3.3.2 Analysis of indirect effects and serial mediation effects

To further elucidate the psychological mechanisms underlying the association between achievement goal orientations and DL, the bootstrap method was used to examine the indirect effects.

The results indicated that, after controlling for the shared variance among the three achievement goal orientations, all three goal orientations exerted significant indirect effects on DL through POF and PC.

Specifically, the total indirect effect of MGO on DL was significant (Effect = 0.228, SE = 0.018, 95% CI [0.193, 0.265]). The total indirect effect of performance-approach goal orientation (PAPG) on DL was also significant (Effect = 0.120, SE = 0.014, 95% CI [0.094, 0.149]). Although the indirect effect of performance-avoidance goal orientation (PAVG) on DL was relatively small, it remained statistically significant (Effect = 0.038, SE = 0.008, 95% CI [0.024, 0.055]).

Further analyses of the specific pathways revealed that POF and PC constituted important psychological pathways through which achievement goal orientations were associated with DL. Specifically, MGO influenced DL through the serial pathway of “POF → PC,” with a serial indirect effect of 0.042 (95% CI [0.031, 0.054]). The corresponding serial indirect effect for PAPG was 0.023 (95% CI [0.014, 0.034]), whereas the serial indirect effect for PAVG was 0.010 (95% CI [0.003, 0.019]).

These findings suggest that achievement goal orientations may first influence physical education teachers’ perceptions of their fit with the organizational environment, which may subsequently facilitate the development of positive coping resources and ultimately contribute to the improvement of DL (Table 4).

**Table 4:** Indirect effects of achievement goal orientations on digital literacy.

Effect pathway	Effect	SE	LLCI	ULCI
MGO				
Total indirect effect	0.228	0.018	0.193	0.265
MGO → POF → PC → DL	0.042	0.006	0.031	0.054
PAPG				
Total indirect effect	0.120	0.014	0.094	0.149
PAPG → POF → PC → DL	0.023	0.005	0.014	0.034
PAVG				
Total indirect effect	0.038	0.008	0.024	0.055
PAVG → POF → PC → DL	0.010	0.004	0.003	0.019

#### 3.3.3 Variance explained by the model

Further analysis of the explanatory power of the model showed that the joint structural model adequately explained the variance in POF, PC, and DL. Specifically, the model explained 41.1% of the variance in POF (*R^2^* = 0.411), 35.4% of the variance in PC (*R^2^* = 0.354), and 40.8% of the variance in DL (*R^2^* = 0.408).

## 4 Discussion

### 4.1 Direct associations between achievement goal orientations and digital literacy among primary and secondary school physical education teachers

First, MGs were significantly and positively associated with the DL of PE teachers. According to the achievement goal theory, individuals with strong MGs tend to exhibit a strong desire to acquire knowledge and improve competence. This intrinsic motivation may encourage physical education teachers to proactively learn digital technologies and technology-supported teaching approaches, thereby serving as an important motivational source for the development of their DL (35). From a cognitive-behavioral perspective, individuals with strong MGs also tend to report higher levels of self-efficacy (36). They are therefore more motivated to acquire technological skills and enhance their professional competence, which may contribute to higher levels of DL (37). Second, teachers with strong PAGs tend to place greater emphasis on demonstrating their competence and obtaining favorable evaluations from colleagues, students, and school administrators. In the context of educational digital transformation, the ability to use digital technologies has increasingly become an important indicator of teachers’ instructional competence and professional performance. To maintain a competitive advantage and gain organizational recognition, these teachers may actively engage in learning digital technologies (38). However, unlike MGs, which are primarily driven by intrinsic motives for learning and competence development, PAGs are more strongly shaped by external evaluation and the need to demonstrate competence. Their association with DL may therefore be more dependent on the specific organizational and evaluative context. Finally, PAvGs were not directly associated with the DL of PE teachers. Teachers with strong PAvGs may fear making operational errors or revealing insufficient competence when using digital technologies. Consequently, they may avoid challenging digital teaching tasks, which may hinder the sustained development of their digital knowledge and application skills (39). However, when schools actively promote educational digitalization and incorporate digital technology use into teachers’ instructional responsibilities, the desire to avoid falling behind or receiving negative evaluations may also motivate teachers to acquire the basic digital skills required to meet job expectations. PAvGs may therefore generate two opposing tendencies: avoiding digital technologies and passively adapting to organizational digitalization requirements. These countervailing effects may explain why their direct association with DL was not significant.

### 4.2 The mediating role of person–organization fit

The findings showed that MGs, PAGs, and PAvGs were all indirectly associated with the DL of PE teachers through POF. First, teachers with strong MGs place greater emphasis on knowledge acquisition, skill development, and professional growth. When schools prioritize educational digitalization and incorporate digital competence into teachers’ professional development requirements, these teachers may be more likely to regard organizational goals as an important direction for their own professional development. They may therefore proactively adjust their knowledge structures and competence profiles to meet schools’ digital development needs. Previous research has shown that greater congruence between teachers’ career values and school characteristics is associated with higher perceived POF (40). In addition, digital training, technological resources, and professional support can satisfy teachers’ competence-development needs and strengthen value congruence, demands–abilities fit, and needs–supplies fit (41). A higher level of POF may further reinforce teachers’ identification with their schools’ digitalization goals, increase their willingness to learn and apply digital technologies, and ultimately promote DL (42). Second, teachers with strong PAGs tend to be more concerned with demonstrating competence, achieving favorable performance, and receiving positive evaluations from others (17). As digital competence becomes increasingly embedded in professional standards, continuing education, and career development systems, these teachers may pay greater attention to their schools’ expectations for digital teaching. They may also demonstrate their competence by learning digital technologies, participating in professional training, and implementing digitally supported physical education. As their digital competence increasingly corresponds to organizational job requirements, demands–abilities fit is likely to improve. At the same time, organizational recognition and rewards for digital teaching outcomes may satisfy their need for affirmation and competence demonstration, thereby enhancing needs–supplies fit. A stronger POF may help translate their motivation to perform well into sustained digital learning and practice, thereby contributing to the development of DL (43). Finally, physical education teachers with strong PAvGs tend to fear work-related mistakes, the exposure of insufficient competence, and negative evaluations. Their instructional behavior may therefore be more strongly influenced by failure avoidance and external evaluative pressure. When confronted with school digitalization initiatives and technology-use requirements, these teachers may adjust their competence and behavior to avoid falling behind their colleagues or failing to meet job expectations. Clear digital standards, accessible technological resources, targeted training, and administrative support can enhance teachers’ perceived organizational support and digital technology self-efficacy, thereby facilitating sustained technology integration (43). Such support may reduce the discrepancy between teachers’ existing competence and organizational expectations, strengthen demands–abilities fit and needs–supplies fit, and gradually encourage teachers to accept and apply digital technologies, ultimately improving their DL (42).

### 4.3 The mediating role of positive coping

This study found that MGO and PAGs were indirectly associated with DL among primary and secondary school physical education teachers through PC, whereas PAvGs were not significantly associated with PC. These findings suggest that different types of achievement goal orientations may differ in their relationships with teachers’ adoption of positive coping strategies. First, mastery-oriented individuals are generally more likely to adopt PC strategies (44). Individuals with strong MGs tend to interpret failure as reflecting insufficient effort or ineffective strategy selection rather than a lack of fixed personal ability. Consequently, they are more likely to adopt adaptive strategies when confronted with difficulties (45). When facing the challenges associated with educational digital transformation, mastery-oriented teachers may therefore respond proactively and continuously improve their digital knowledge and technological application skills. Previous studies have also shown that PAGs are positively associated with help-seeking when individuals encounter difficulties and challenges. Individuals with strong PAGs may be simultaneously motivated by the pursuit of success and the avoidance of failure (46). To perform better than others, PE teachers may therefore adopt PC strategies when facing digital teaching challenges and use these strategies to improve their DL. Second, this study found that PAvGs did not significantly predict PC. This finding is consistent with previous research suggesting that performance-avoidance goals may be associated with maladaptive outcomes (47). Achievement goal theory posits that performance-avoidance goals are typically characterized by a desire to avoid failure, concerns about negative evaluation, and a tendency to minimize risks, which may reduce individuals’ willingness to adopt proactive strategies when facing challenges (47). In the context of digital teaching, physical education teachers with higher levels of PAvG may focus more on avoiding operational errors and reducing evaluation risks, rather than proactively adopting positive coping strategies such as learning, exploration, and problem solving.

### 4.4 The serial mediating roles of person–organization fit and positive coping

The findings showed that MGs, PAGs, and PAvGs were all associated with the DL of PE teachers through the serial pathway of POF and PC. First, value congruence may strengthen physical education teachers’ identification with their schools’ digital development philosophy and digital teaching goals. When teachers’ professional development aspirations are aligned with their schools’ digitalization strategies, they are more likely to interpret digital technology use as an important means of improving teaching and advancing professional growth rather than as a purely external requirement. Previous research has shown that higher levels of fit are closely associated with positive work attitudes and work engagement, and recent evidence has indicated that POF significantly predicts employee work engagement (48). In digital education contexts, school digital policies and culture, together with the satisfaction of teachers’ basic psychological needs, may also encourage their participation in digital competence development activities (41). Higher value congruence may therefore provide an important foundation for identification and intrinsic motivation, making physical education teachers more willing to respond to digital teaching difficulties through active learning, problem solving, and support seeking. Second, demands–abilities fit may enhance teachers’ sense of competence and control when completing digital teaching tasks. When teachers’ digital knowledge and skills correspond to their schools’ expectations for digital instruction, they are more likely to evaluate their own ability to complete technology-related tasks positively and to regard digital teaching requirements as manageable challenges. Empirical research has also shown that ICT self-efficacy mediates the relationship between perceived school support and sustained technology integration among teachers (45). Accordingly, stronger demands–abilities fit may reduce uncertainty arising from perceived competence deficits and make physical education teachers more likely to engage in repeated practice, strategy adjustment, proactive help-seeking, and problem solving. Finally, needs–supplies fit may provide the organizational resources required for teachers to cope positively with digital teaching challenges. When schools provide digital equipment, technological training, sufficient time, peer assistance, and professional development opportunities that correspond to teachers’ actual needs, teachers not only gain the external conditions necessary to address digital teaching problems but are also more likely to perceive organizational support. A three-wave longitudinal study found that organizational support enhanced employees’ needs–supplies fit and subsequently promoted proactive skill development (49). Research involving physical education teachers has further identified insufficient technological resources, inadequate training, limited time, and weak organizational support as major barriers to digital technology use (50). Therefore, when organizational provisions are well aligned with teachers’ needs, teachers have access to more coping resources and may be more likely to use these resources proactively, collaborate with colleagues, and engage in continuous learning.

Overall, AGOs may shape the extent to which PE teachers adjust their values, competence, and developmental needs to accommodate the digital environment of their schools. Higher levels of value congruence, demands–abilities fit, and needs–supplies fit may promote PC by strengthening goal identification, competence beliefs, and resource availability, respectively. PC, in turn, may enable teachers to translate their achievement-related motivation into active learning and the application of digital technologies, ultimately enhancing their DL.

## 5 Conclusion

This study developed and validated a psychological mechanism model of DL development among primary and secondary school PE teachers based on POF theory and achievement goal theory. Different achievement goal orientations showed distinct patterns in their associations with digital literacy. Specifically, MGO and PAGs were not only indirectly associated with DL through the serial pathway involving POF and PC but also demonstrated significant direct effects, whereas PAvGs did not exhibit a significant direct predictive effect. These findings further elucidate the mechanism underlying the development of teachers’ digital literacy, characterized by the pathway of “motivation–cognitive appraisal–behavioral regulation–competence development.”

POF and PC play sequential roles in this process. Specifically, POF reflects PE teachers’ cognitive evaluation of the compatibility between themselves and the school’s digital environment, whereas PC represents their proactive behavioral strategies in responding to challenges associated with digital teaching. Together, these two processes constitute an important psychological and behavioral mechanism through which motivational characteristics are transformed into DL.

By distinguishing different AGOs, this study further reveals the motivational heterogeneity underlying DL development among PE teachers and extends the application of achievement goal theory to the field of teacher DL research. These findings suggest that schools should consider individual differences in teachers’ motivational orientations when promoting DL development and facilitate sustainable digital competence development by optimizing organizational support and fostering PC.

### 5.1 Limitations and future directions

Several limitations should be acknowledged. First, the cross-sectional design of this study only permits the identification of associations among the variables and does not allow for rigorous causal inference. Future research could employ longitudinal designs or randomized intervention studies to examine the dynamic and potentially causal relationships among AGDs, POF, PC, and the DL of PE teachers.

Second, this study focused primarily on the serial mediating roles of POF and PC. Other psychological mechanisms that may influence teachers’ DL, such as self-efficacy and perceived behavioral control over technology use, were not included. Future studies could develop more comprehensive theoretical models incorporating these factors to provide a more complete explanation of how AGDs are associated with the DL of physical education teachers. Meanwhile, because this study used an anonymous survey approach, school identification information was not collected; therefore, potential clustering effects arising from teachers within the same school could not be further examined. Future studies should incorporate additional individual- and organizational-level variables and employ multilevel analysis to further elucidate the mechanisms underlying DL development among primary and secondary school PE teachers.

Third, all study variables were measured using teacher self-reports. The findings may therefore have been affected by common method bias and social desirability. Future research could integrate multiple sources of data, including classroom observations, digital teaching-platform usage records, evaluations of digitally supported teaching products, and assessments provided by school administrators, colleagues, and students. Such multimethod approaches would improve the objectivity, reliability, and ecological validity of the findings.

Fourth, this study adopted a convenience sampling method, and the sample was primarily drawn from primary and secondary school PE teachers in Guangdong Province, China. Although the relatively large sample size enhanced the stability of the statistical analyses, convenience sampling may limit the representativeness of the sample. Furthermore, Guangdong Province is characterized by a relatively high level of economic development and more advanced educational digitalization compared with many other regions in China. These contextual advantages may have positively influenced teachers’ access to digital resources and their perceptions of POF. Therefore, future research should employ more diverse sampling strategies and conduct cross-regional validations to enhance the external validity and generalizability of the findings.

## Data Availability

The datasets used and/or analyzed during the current study are available from the corresponding author on reasonable request. Requests to access the datasets should be directed to zhang.sfeng@163.com.
